# Health care experiences during the COVID-19 pandemic by race and social determinants of health among adults age ≥ 58 years in the REGARDS study

**DOI:** 10.1186/s12889-021-12273-8

**Published:** 2021-12-11

**Authors:** Emily B. Levitan, Virginia J. Howard, Mary Cushman, Suzanne E. Judd, Stephanie E. Tison, Ya Yuan, Debora Kamin Mukaz, Henry E. Wang, Nathalie Pamir, Timothy B. Plante, Stephen P. Juraschek, Monika M. Safford, Parag Goyal

**Affiliations:** 1 School of Public Health, University of Alabama at Birmingham, Birmingham, AL 35294-0022 USA; 2 Department of Medicine, Larner College of Medicine at the University of Vermont, Burlington, VT USA; 3 Department of Emergency Medicine, The Ohio State University, Columbus, OH USA; 4 Oregon Health and Science University, Portland, OR USA; 5 Beth Israel Deaconess Medical Center, Boston, MA USA; 6 Weill Cornell Medicine, New York, NY USA

**Keywords:** COVID-19, Social determinants of health, Health care access

## Abstract

**Background:**

Understanding health care experiences during the COVID-19 pandemic may provide insights into patient needs and inform policy. The objective of this study was to describe health care experiences by race and social determinants of health.

**Methods:**

We conducted a telephone survey (July 6, 2020-September 4, 2021) among 9492 Black and White participants in the longitudinal REasons for Geographic And Racial Differences in Stroke cohort study, age 58–105 years, from the continental United States. Among participants with symptoms of COVID-19, outcomes were: 1. Sought care or advice for the illness; 2. Received a SARS-CoV-2 test for the illness; and 3. Tested positive. Among participants without symptoms of COVID-19, outcomes were: 1. Wanted a test; 2. Wanted and received a test; 3. Did not want but received a test; and 4. Tested positive. We examined these outcomes overall and in subgroups defined by race, household income, marital status, education, area-level poverty, rural residence, Medicaid expansion, public health infrastructure ranking, and residential segregation.

**Results:**

The average age of participants was 76.8 years, 36% were Black, and 57% were female. Among participants with COVID-19 symptoms (*n* = 697), 74% sought care or advice for the illness, 50% received a SARS-CoV-2 test, and 25% had a positive test (50% of those tested). Among participants without potential COVID-19 symptoms (*n* = 8795), 29% wanted a SARS-CoV-2 test, 22% wanted and received a test, 8% did not want but received a test, and 1% tested positive; a greater percentage of participants who were Black compared to White wanted (38% vs 23%, *p* < 0.001) and received tests (30% vs 18%, *p* < 0.001) and tested positive (1.4% vs 0.8%, *p* = 0.005).

**Conclusions:**

In this national study of older US adults, many participants with potential COVID-19 symptoms and asymptomatic participants who desired testing did not receive COVID-19 testing.

**Supplementary Information:**

The online version contains supplementary material available at 10.1186/s12889-021-12273-8.

## Background

People of color in the United States have been disproportionately affected by the COVID-19 pandemic [1–4]. Black individuals have an age-adjusted 10% higher rate of COVID-19, 3.8-fold higher rate of hospitalization, and 2.0-fold higher rate of mortality compared to White individuals [5]. These disparities in COVID-19 and its attendant mortality are hypothesized to arise from multiple levels of racism which lead to disparities in social determinants of health (SDOH), characteristics of individuals’ social and physical environments that contribute to health inequities [6–13]. Racism and SDOH amplify the impact of the COVID-19 pandemic on vulnerable populations through greater risk of exposure, greater prevalence of factors that increase the risk of severe disease, and less access to appropriate health care [2, 3, 9, 10, 13–18]. Black individuals are more likely to be uninsured and to face barriers to accessing health care [8, 9], and structural and implicit bias may lead to inequitable provision of health care and worse outcomes [19].

Several studies have examined rates of SARS-CoV-2 testing by race/ethnicity using data from health systems [20, 21]. However, data are sparse on the health care experience during the COVID-19 pandemic from the patient perspective. Understanding the role of race and SDOH in experiences of care may identify subpopulations at greatest risk during the COVID-19 pandemic and future pandemics, and inform patient needs and community- and national-level policies to protect the most vulnerable people. The REasons for Geographic and Racial Differences in Stroke (REGARDS) cohort study, which includes community-dwelling Black and White adults from across the continental United States (US) recruited in 2003–2007 with current mean age 77 years (range 58–105), collected data on SDOH prior to the COVID-19 pandemic [22]. Beginning in the summer of 2020, during semi-annual follow-up, the REGARDS participants completed surveys about their experiences with SARS-CoV-2 testing and changes in access to care. The objective of this study was to describe patterns in experiences with health care during the COVID-19 pandemic by race and SDOH.

## Methods

### Study population

The study population was drawn from participants in the REGARDS study, details of which have been previously published [22]. In brief, REGARDS was designed to investigate the reasons for high rates of stroke mortality among Black Americans and individuals in the Southeastern US. In 2003–2007, 30,239 participants from the 48 contiguous states of the US were identified from commercial lists and recruited using mailings and telephone calls, with oversampling of Black individuals and individuals living in the Southeastern US. Individuals were eligible if they self-identified as Black or White race, non-Hispanic ethnicity, and age 45 years or older. The telephone response rate was 33% and the cooperation rate was 49% [23]. Participants completed a computer assisted telephone interview (CATI) and brief in-home physical exam at baseline and received $30 US as an incentive [22]. Approximately 10 years after baseline (2013–2016), 16,146 participants (63% of surviving Black participants and 72% of surviving White participants) completed a second computer assisted telephone interview and in-person assessment [24]; the incentive for the second in-person assessment was up to $100 US. The data collection instruments, available at https://www.uab.edu/soph/regardsstudy/, were developed for the REGARDS study, adapted from widely used surveys. Since recruitment, participants have been interviewed every 6 months for assessment of health events and cognitive function. Beginning July 6, 2020, a survey module was added to the 6-month follow-up calls to assess SARS-CoV-2 testing, COVID-19 illness, and experiences of health care during the pandemic (Additional file 1). At that time, there were approximately 11,000 participants still in active follow-up, mean age 76.9 years (range 58–105). The current analysis included participants who completed this survey module as of September 4, 2021. For participants who completed the survey module more than once, we selected the first survey completed.

The REGARDS study was approved by institutional review boards (IRB) at the participating institutions. All participants provided written informed consent. In order to abide by its obligations with the National Institutes of Health National Institute of Neurologic Disorders and Stroke (NIH/NINDS) and the IRB of the University of Alabama at Birmingham, REGARDS facilitates data sharing through formal data use agreements. Any investigator is welcome to request the REGARDS data and documentation through this process. Requests for data access may be sent to the REGARDS study at regardsadmin@uab.edu.

### Assessment of SDOH

As in previous publications in the REGARDS study population, we evaluated SDOH in the domains described in the Healthy People 2020 Framework [12, 25]. Specifically, we anticipated that less than high school education, being unmarried, annual household income <$35,000, residence in a ZIP code (US postal code) with > 25% of individuals below the federal poverty line, rural residence, living in a state without Medicare expansion or with limited public health infrastructure and greater residential segregation would be associated with less access to health care including SARS-CoV-2 testing. Education was reported by participants during the baseline telephone interview. Annual household income and marital status were self-reported during the baseline and second interviews. We used responses from the second interview, except for those participants missing data for whom the first interview responses were included (income: *n* = 341; marital status: *n* = 389). As in prior work, we included an income “not reported category” because a substantial proportion of participants declined to report income [25]. Participant residential addresses at baseline and the second assessment were geocoded using ArcGIS and a minimum match score of 90; the match rate was 97% [26]. The distribution of REGARDS participants’ addresses has been described in a previous publication [23]. We used the 2010 Rural Urban Commuting Area (RUCA) codes to identify those living in rural areas, except for individuals for whom address was not available at the second assessment [27]. For these 306 participants, rurality was defined from the first visit using the 2000 RUCA classification [27]. For measures of Medicaid expansion and public health infrastructure, addresses at the second in-home assessment were used, except for 417 participants who had available geocoded addresses at the first but not second assessments. At the state level, we examined whether the participant lived in a state that did not expand Medicaid (Alabama, Florida, Georgia, Kansas, Mississippi, North Carolina, South Carolina, South Dakota, Tennessee, Texas, Wisconsin, Wyoming) and whether the participant lived in a state with poor public health infrastructure (Arkansas, Florida, Louisiana, New Mexico, Mississippi, Nevada, South Carolina, Texas, Tennessee). Public health infrastructure was based on America’s Health Rankings, a state-level composite measure of public health status [28]. As in prior work, states in the bottom 20th percentile of the ranking for 8 or more years during the ten-year period 1993–2002 were considered to have poor public health infrastructure [25]. Residential segregation was assessed using the dissimilarity index from the geocoded address at baseline with information from the 2000 US Census [29]. The dissimilarity index ranges from 0 to 1, where larger values indicate a greater degree of residential segregation. For presentation, the dissimilarity index was dichotomized at the median value (0.50). ZIP code level poverty was also assessed using baseline participant address and information from the 2000 US Census.

### Assessment of other characteristics

Date of birth was reported during the baseline telephone interview and used to calculate age at the time of survey administration. Hypertension was defined as systolic blood pressure ≥ 140 mmHg, diastolic blood pressure ≥ 90 mmHg, or self-report of use of antihypertensive medications at baseline or the 10-year follow-up visit. Diabetes was defined at baseline or 10-year follow-up as fasting blood glucose ≥126 mg/dl (or a glucose ≥200 mg/dl among those who did not fast) or self-report of use of glucose lowering medications. History of stroke, coronary heart disease, and heart failure were based on self-report of physician diagnoses at baseline or the 10-year follow-up or clinician-adjudicated events during follow-up [23, 30, 31]. Cigarette smoking and body mass index were assessed at the 10-year follow-up visit; baseline values were used for 428 participants missing follow-up visit information on cigarette smoking and 709 participants missing follow-up visit information on body mass index.

### Health care experiences during the COVID-19 pandemic

The survey module included early questions from the Centers for Disease Control and Prevention and the National Institute of Health recommended common data elements for COVID-19, adapted for telephone administration (Additional file 1). The time referent for the survey was the prior 6 months. Additional file 2 Fig. 1 illustrates the survey logic. Among participants who reported an illness with symptoms of COVID-19 (fever, cough sore throat, chills, muscle pain, loss of taste or smell, or shortness of breath or difficulty breathing), we examined the following outcomes: 1. Sought care or advice for the illness; 2. Received a SARS-CoV-2 test for the illness; and 3. Tested positive. Among participants who did not report an illness with symptoms of COVID-19, we examined the following outcomes: 1. Wanted a SARS-CoV-2 test; 2. Wanted and received a SARS-CoV-2 test; 3. Did not want but received a SARS-CoV-2 test; and 4. Tested positive. Among all participants, we examined participant characteristics by self-reported subjective COVID-19 and self-reported positive SARS-CoV-2 test. Additionally, we examined the impact of the pandemic on access to health care; survey options were “No change”, “Mild. Appointments moved to telehealth”, “Moderate. Delays or cancellations in appointments and/or delays in getting prescriptions; changes have minimal impact on health.”, and “Severe. Unable to access needed care resulting in moderate to severe impact on health.”

### Statistical analysis

We calculated the percentage of participants experiencing each of the COVID-19 related outcomes overall and by race and each of the SDOH. We used chi-square tests for categorical variables and ANOVA for continuous variables to evaluate whether differences between groups were statistically significant. Participants missing data on a characteristic were excluded from the analysis for that characteristic. For each variable, data were missing for < 5% of participants. In secondary analyses, we standardized the population to the age, race, sex, and geographic distribution from the US Census 2020 estimated population age 58 years or older residing in the 48 contiguous states, who were not Hispanic, and who reported Black or White race [32]. Strata for calculating weights were defined by age (58–74, 75–84, and ≥ 85 years), sex, race, and Southeastern states (Alabama, Arkansas, Georgia, Louisiana, Mississippi, North Carolina, South Carolina, and Tennessee) versus others. Statistical significance of differences between groups for standardized estimates were tested using bivariate logistic regression with inverse probability of sampling weights and robust standard errors. Additionally, we stratified the study population by calendar year of survey administration (2020 or 2021) and tested for differences across years using chi-square tests. Analyses were performed using SAS version 9.4 (Cary, NC) and Stata version 17.0 (College Station, TX). Two-sided *p*-values < 0.05 were considered statistically significant.

## Results

### Study population

Among 9492 participants who completed the REGARDS COVID-19 survey module, the average age was 76.8 years (range 58–105 years), 92% were age 65 years or older, 36% were Black, and 57% were female (Table 1). Participants resided in 1279 counties across the 48 continental United States. Thirty percent of participants had income <$35,000 per year, 6% had less than high school education, 39% were unmarried, 16% lived in ZIP code areas where > 25% of residents were below the federal poverty limit, 17% lived in rural areas, 33% lived in states with poor public health infrastructure, and 54% lived in states that did not expand Medicaid. Illnesses with COVID-19 symptoms were reported by 697 participants (7%). Participants who reported illness with COVID-19 symptoms were slightly younger, had higher body mass index, and more commonly had diabetes and history of coronary heart disease compared to participants who did not report such an illness.

**Table 1:** Characteristics of REGARDS participants by self-report of illness with COVID-19 symptoms^a^

	Overall (***N*** = 9492)	Illness with COVID-19 symptoms	
		Yes (***N*** = 697)	No (***N*** = 8795)
Age, y	76.8 **±** 7.8	74.8 ± 7.3	77.0 ± 7.8
Sex			
Male	4041 (42.6%)	278 (39.9%)	3763 (42.8%)
Female	5451 (57.4%)	419 (60.1%)	5032 (57.2%)
Race			
Black	3416 (36.0%)	238 (34.1%)	3178 (36.1%)
White	6076 (64.0%)	459 (65.9%)	5617 (63.9%)
Income < $35,000	2876 (30.3%)	216 (31.0%)	2660 (30.2%)
Unmarried	3725 (39.2%)	257 (36.9%)	3468 (39.4%)
Less than high school education	558 (5.9%)	39 (5.6%)	519 (5.9%)
Area-level poverty > 25%	1488 (16.0%)	116 (17.0%)	1372 (15.9%)
Rural residence	1584 (16.9%)	133 (19.4%)	1451 (16.7%)
Poor public health infrastructure	3136 (33.0%)	246 (35.3%)	2890 (32.9%)
Medicaid non-expansion state	5131 (54.1%)	372 (53.4%)	4759 (54.1%)
Higher residential segregation	4744 (50.0%)	323 (46.4%)	4421 (50.3%)
Cigarette smoking			
Current	611 (6.4%)	35 (5.0%)	576 (6.6%)
Past	3827 (40.3%)	305 (43.8%)	3522 (40.1%)
Never	5049 (53.2%)	356 (51.2%)	4693 (53.4%)
BMI, kg/m^2^	29.4 **±** 6.4	30.5 **±** 6.8	29.3 **±** 6.4
Diabetes	2155 (22.7%)	196 (28.1%)	1959 (22.3%)
Hypertension	6277 (66.1%)	473 (67.9%)	5804 (66.0%)
History of stroke	574 (6.1%)	46 (6.6%)	528 (6.0%)
History of coronary heart disease	1868 (19.7%)	170 (24.4%)	1698 (19.3%)
History of heart failure	167 (1.8%)	13 (1.9%)	154 (1.8%)

^a^Numbers in table are N (column %) or mean ± SD. There were missing data for education (*n* = 1), area-level poverty (*n* = 166), rural residence (*n* = 101), residential segregation (*n* = 7), cigarette smoking (*n* = 5), BMI (*n* = 6). Data were complete for all other variables

### Participants reporting COVID-19 symptoms

**Fig. 1:**
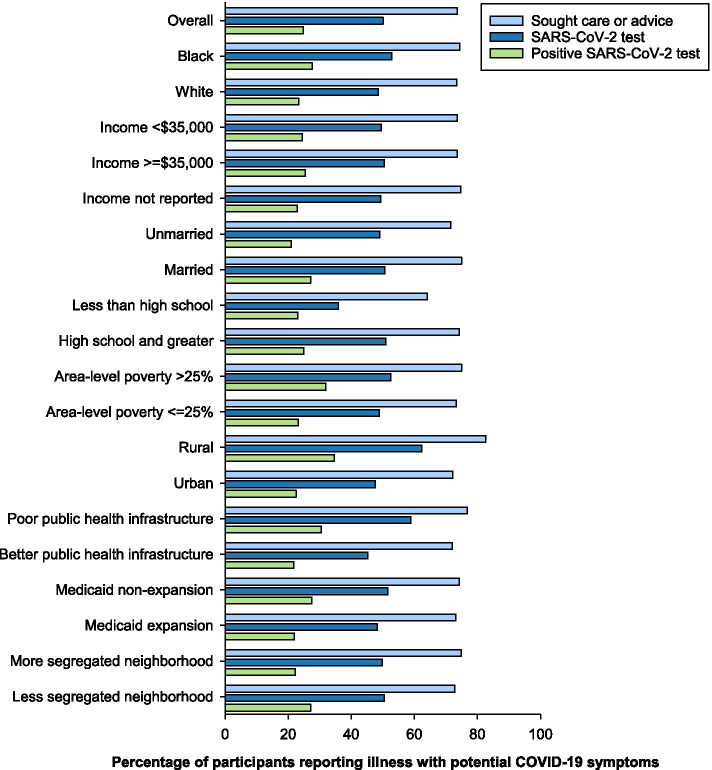
Race, social determinants of health, and SARS-CoV-2 testing among participants in the REasons for Geographic And Racial Differences in Stroke study who reported illness with symptoms of COVID-19

Among the 697 participants reporting an illness with COVID-19 symptoms, 514 (74%) sought care or advice for that illness, 349 (50%) received a SARS-CoV-2 test for that illness, and 173 participants had a positive SARS-CoV-2 test (25%) (Fig. 1 and Additional file 2 Table 1). Of participants tested, 50% (173 of 349) tested positive. Compared to urban residents, a higher percentage of participants residing in rural areas sought care or advice for an illness with COVID-19 symptoms (83% vs 72%, *p* = 0.03), received a SARS-CoV-2 test (62% vs 48%, *p* = 0.002), and tested positive (35% vs 23%, *p* = 0.004). A higher percentage of participants in states with poor public health infrastructure, compared to better, received SARS-CoV-2 testing (59% vs 45%, *p* = < 0.001) and tested positive for SARS-CoV-2 (30% vs 22%, *p* = 0.01). A higher percentage of participants living in areas with > 25% poverty tested positive than participants in lower poverty areas (32% vs 23%, *p* = 0.047). Differences by other characteristics were not statistically significant.

When standardized to the age, sex, and geographic distribution of the US population, a higher percentage of Black compared to White participants received SARS-CoV-2 tests (58% vs 44%, *p* = 0.02) and tested positive (34% vs 20%, *p* = 0.007) (Additional file 2 Table 1a). Compared to participants responding to the survey in 2020, the percentage of participants reporting seeking care or advice in 2021 was slightly higher (77% vs 73%, *p* = 0.03), and the percentage receiving SARS-COV-2 tests (67% vs 44%, *p* < 0.001) and testing positive (47% vs 16%, *p* < 0.001) was substantially higher (Additional file 2 Table 1b and c). Compared to White participants, Black participants reported higher proportion of positive tests in 2020 (22% vs 14%, *p* = 0.01) and lower proportion of positive tests in 2021, though the difference between Black and White participants was not statistically significant in 2021 (40% vs 51%, *p* = 0.14).

### Participants not reporting COVID-19 symptoms

**Fig. 2:**
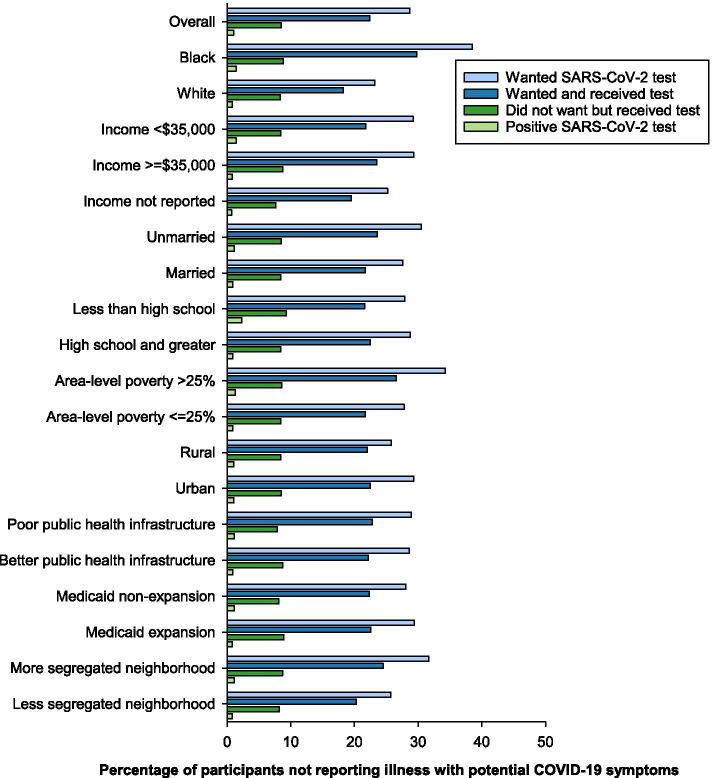
Race, social determinants of health, and SARS-CoV-2 testing among participants in the REasons for Geographic And Racial Differences in Stroke study who did not report illness with symptoms of COVID-19

Of the 8795 participants who did not report an illness with COVID-19 symptoms, 2525 (29%) reported wanting SARS-CoV-2 testing, 1972 (22%) wanted and received a test, and 744 (8%) had a SARS-CoV-2 test despite not reporting wanting one (Fig. 2 and Additional file 2 Table 2). Among the participants who did not report COVID-19 symptoms, 85 had a positive SARS-CoV-2 test (1% of participants not reporting COVID-19 symptoms; 3% of those tested). A higher percentage of Black compared to White participants reported wanting a SARS-CoV-2 test (38% vs 23%, *p* < 0.001), receiving a SARS-CoV-2 test (30% vs 18%, *p* < 0.001), and testing positive compared to White participants (1.4% vs 0.8%, *p* = 0.005). A greater percentage of lower-income participants reported testing positive (1.4% vs 0.8%, *p* = 0.03) compared to higher income participants. Being unmarried, compared to married, was associated with a greater percentage of participants reporting wanting a SARS-CoV2 test (30% vs 28%, *p* = 0.003) and receiving a test (24% vs 22%, *p* = 0.03). Individuals with less than high school education reported testing positive more commonly than individuals with high school education or greater (2.3% vs 0.9%, *p* = 0.001). Rural compared to urban residence was associated with a lower percentage desiring testing (26% vs 29%, *p* = 0.008). Area-level poverty > 25% and greater residential segregation were associated with higher percentage reporting desire and receipt of testing.

Patterns of reporting wanting SARS-CoV-2 tests, receiving tests, and testing positive were similar when standardized to the age, sex, and geographic distribution of the US population (Additional file 2 Table 2a). Compared to participants who completed the survey in 2020, a higher percentage of participants completing the survey in 2021 reported wanting a SARS-CoV-2 test (37% vs 26%, *p* < 0.001), wanting and receiving a test (30% vs 20%, *p* < 0.001), receiving a test despite not wanting one (10% vs 8%, *p* < 0.001), and testing positive (1.5% vs 0.8%, *p* = 0.002) (Additional file 2 Table 2b and c).

### Subjective report of COVID-19

Of the 279 total participants reporting a positive SARS-CoV-2 test, 74 (27%) reported that they did not think they had COVID-19, of whom 23 reported an illness with symptoms of COVID-19. Compared to participants testing positive who thought they had COVID-19 (*n* = 205), participants who did not think they had COVID-19 were more commonly Black, low income, unmarried, and female (Table 2). Among the 9213 participants without a positive SARS-CoV-2 test, 297 reported that they thought they had COVID-19; 100 (34%) of those participants reported an illness with symptoms of COVID-19 and 140 (47%) received SARS-CoV-2 testing.

**Table 2:** Characteristics of REGARDS participants by subjective report of COVID-19 and report of results of testing for SARS-CoV-2^a^

Positive SARS-CoV-2 test	Yes (***N*** = 279)		No (***N*** = 9213)		***P***-value^b^
Subjective report of COVID-19	Yes (***N*** = 205)	No (***N*** = 74)	Yes (***N*** = 297)	No (***N*** = 8916)	
Illness with COVID-19 symptoms	171 (83.4%)	23 (31.1%)	100 (33.7%)	403 (4.5%)	< 0.001
Received SARS-CoV-2 testing	205 (100%)	74 (100%)	140 (47.1%)	2799 (31.4%)	< 0.001
Age, y	73.3 ± 7.1	77.6 ± 7.6	73.0 ± 7.3	77.0 ± 7.8	< 0.001
Sex					0.01
Male	91 (44.4%)	25 (33.8%)	102 (34.3%)	3823 (42.9%)	
Female	114 (55.6%)	49 (66.2%)	195 (65.7%)	5093 (57.1%)	
Race					0.003
Black	76 (37.1%)	42 (56.8%)	105 (35.4%)	3193 (35.8%)	
White	129 (62.9%)	32 (43.2%)	192 (64.6%)	5723 (64.2%)	
Income < $35,000	69 (33.7%)	30 (40.5%)	92 (31.0%)	2685 (30.1%)	0.23
Unmarried	67 (32.7%)	36 (48.7%)	115 (38.7%)	3507 (39.3%)	0.09
Less than high school education	12 (5.9%)	10 (1.8%)	18 (6.1%)	518 (5.8%)	0.048
Area-level poverty > 25%	48 (23.8%)	12 (16.9%)	45 (15.4%)	1383 (15.8%)	0.02
Rural residence	48 (23.9%)	15 (20.5%)	52 (17.8%)	1469 (16.6%)	0.04
Poor public health infrastructure	81 (39.5%)	28 (37.8%)	105 (35.4%)	2922 (32.8%)	0.13
Medicaid non-expansion state	112 (54.6%)	51 (68.9%)	150 (50.5%)	4818 (54.0%)	0.04
Higher neighborhood segregation	94 (45.9%)	37 (50.0%)	158 (53.2%)	4455 (50.0%)	0.45
Cigarette smoking					0.56
Current	13 (6.4%)	6 (8.1%)	15 (5.1%)	557 (6.5%)	
Past	90 (44.1%)	29 (39.2%)	109 (36.7%)	3599 (40.4%)	
Never	101 (49.5%)	39 (52.7%)	173 (58.3%)	4736 (53.1%)	
BMI, kg/m^2^	30.8 ± 7.4	30.3 ± 6.2	30.7 ± 6.8	29.3 ± 6.4	< 0.001
Diabetes	63 (30.7%)	18 (24.3%)	68 (22.9%)	2006 (22.5%)	0.049
Hypertension	134 (65.4%)	53 (71.6%)	184 (62.0%)	5906 (66.2%)	0.33
History of stroke	13 (6.3%)	4 (5.4%)	18 (6.1%)	539 (6.1%)	0.99
History of coronary heart disease	43 (21.0%)	16 (21.6%)	60 (20.2%)	1749 (19.6%)	0.93
History of heart failure	2 (1.0%)	3 (4.1%)	5 (1.7%)	157 (1.8%)	0.39

^a^Numbers in table are N (column %) or mean ± SD

^b^Comparison across 4 groups defined by positive SARS-CoV-2 and subjective report of COVID-19, using chi-squared tests for categorical variables and ANOVA for continuous variables

### Changes in access to care

**Fig. 3:**
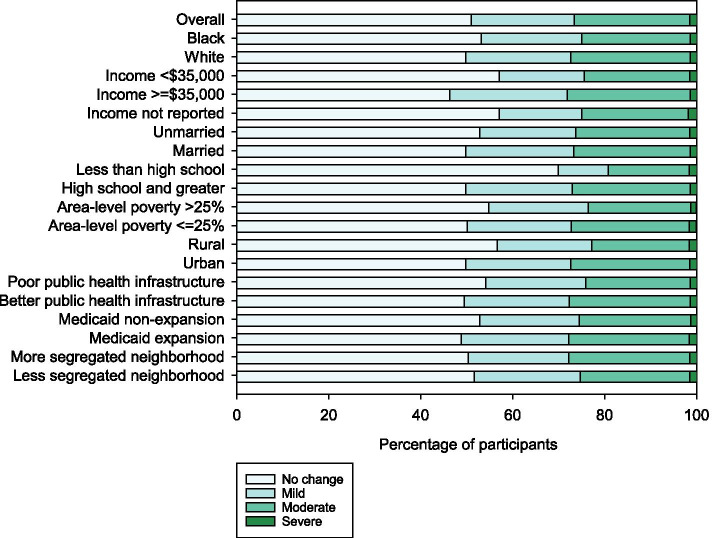
Social determinants of health and impact of the COVID-19 pandemic on access to care among participants in the REasons for Geographic And Racial Differences in Stroke study. Survey options were “No change”, “Mild. Appointments moved to telehealth”, “Moderate. Delays or cancellations in appointments and/or delays in getting prescriptions; changes have minimal impact on health.”, and “Severe. Unable to access needed care resulting in moderate to severe impact on health”

Overall, 51% of participants reported no change in access to care during the COVID-19 pandemic, 22% reported mild changes, 25% reported moderate changes, and 1% reported severe changes (Fig. 3 and Additional file 2 Table 3). Reporting no change in access to care was more common in Black than White participants (53% vs 50%, *p* = 0.01), those with income <$35,000 compared to ≥$35,000 (57% vs 46%, *p* < 0.001), unmarried compared to married participants (53% vs 50%, *p* = 0.005), individuals with less than high school education compared to high school or greater (70% vs 50%, *p* < 0.001), residence with area-level poverty > 25% compared to ≤25% (55% vs 50%, *p* = 0.006), rural compared to urban (57% vs 50%, *p* < 0.001), those in states with poor public health infrastructure compared to better public health infrastructure (54% vs 50%, *p* < 0.001), and Medicaid non-expansion states compared to Medicare expansion states (53% vs 49%, *p* = 0.002). Patterns were similar when standardized to the age, race, sex, and geographic distribution of the US (Additional file 2 Table 3a). A greater percentage of participants who completed the survey in 2021 reported no change (56%) compared to those who completed the survey in 2020 (49%, *p* < 0.001) (Additional file 2 Table 3b and c).

## Discussion

In this large, national US sample of Black and White older adults from the geographically diverse REGARDS cohort, our results showed several important findings regarding experiences with health care during the COVID-19 pandemic. First, only 74% of participants who had symptoms consistent with COVID-19 sought care or advice for that illness and only 50% received a SARS-CoV-2 test. These patterns were largely consistent across race and a broad range of characteristics reflecting participants’ social and physical environment. Among those participants who did not report COVID-19 symptoms, nearly a quarter of participants who wanted SARS-CoV-2 testing did not receive it. This lack of testing has likely contributed to under-ascertainment and subsequently underestimation of COVID-19 illness and SARS-CoV-2 infection. Additionally, nearly one third of participants who had a positive test for SARS-CoV-2 reported that they did not think they had COVID-19. Finally, a greater percentage of participants who were Black, had income <$35,000, were unmarried, and had less than high school education reported no change in usual health care received during the pandemic compared to participants without these characteristics. A greater percentage of participants who lived in a rural area, high-poverty area, a state with poor public health infrastructure, or Medicaid non-expansion state reported no change in usual health care received during the pandemic as compared to participants not living in these areas.

In this study, a substantial proportion of participants who were symptomatic did not receive testing for SARS-CoV-2, particularly among participants responding to the survey in 2020. Early in the pandemic when many hospitals were overwhelmed with severe cases of COVID-19 and testing capacity was extremely limited, the US Centers for Disease Control and Prevention recommended that individuals with mild symptoms self-isolate and that testing be prioritized for hospitalized patients, high-risk individuals, and health care workers [33]. However, confirmation of SARS-CoV-2 infection can promote improved adherence to infection control behaviors such as handwashing and mask-wearing, as well as isolation of infected persons and self-quarantine of contacts. While there were some differences in receipt of SARS-CoV-2 testing by race and SDOH, our findings suggest that barriers to testing were experienced broadly. Importantly, the overall dearth of SARS-CoV-2 testing may have had a disproportionately negative impact on higher-risk populations in which the percentage of test positivity was higher, including Black Americans [34]. Our finding that Black participants were more likely to receive testing and report positive SARS-CoV-2 tests, consistent with prior reports [17, 20, 21, 35, 36], raises the possibility of a high prevalence of underreporting of symptoms and undetected SARS-CoV-2 infection and COVID-19 in Black persons. Studies examining patterns in SARS-CoV-2 antibody formation will provide a greater understanding of the scope of the pandemic, and the differential impact of limited testing on vulnerable populations.

We found that nearly a third of participants who reported a positive SARS-CoV-2 test reported that they did not think that they had COVID-19. Our survey did not distinguish between symptomatic COVID-19 illness and the causative virus SARS-CoV-2. While many of these individuals likely had asymptomatic infections [37], these findings may also in part reflect skepticism and distrust. Understanding sources of skepticism and distrust and developing strategies to mitigate them is important for effective pandemic mitigation efforts and vaccination campaigns.

Contrary to our expectations, we found that Black individuals and those with SDOH associated with barriers to health care more commonly reported no change in access to medical care related to the pandemic as compared to White individuals and individuals without these SDOH. This finding may reflect limited access to care prior to the pandemic, such that the pandemic did not yield a discernible effect. This potential explanation is supported by a prior report that residents of ZIP codes with lower (compared to higher) income and with higher (compared to lower) racial or ethnic minority populations had less health care utilization prior to the pandemic and experienced smaller changes in health care utilization during the pandemic [38].

This study primarily included older adults who nearly all had health insurance and who had been enrolled in a longitudinal health research study for over a decade, which limits the generalizability of the findings. Age and health insurance may modify the associations of race and SDOH with experiences during the pandemic. More than 90% of the participants in the REGARDS study were aged 65 years or older at the time of survey administration and therefore eligible for Medicare coverage, a federal health insurance program for older US adults. Indeed, few REGARDS participants report being uninsured [39]. Many older adults have established relationships with health care professionals facilitating access to care throughout the pandemic. Additionally, long-term participation in a health research study may reflect greater access to and engagement with health care resources. Future studies should examine racial and SDOH-related disparities in experiences of health care during the COVID-19 pandemic among younger populations and uninsured populations.

Strengths of this study include the large population of Black and White adults who reported their experiences of health care during the COVID-19 pandemic. Participants were from a large number of counties across the US and had pre-pandemic assessment of SDOH. There are also several limitations. These analyses relied solely on self-reported information regarding COVID-19 symptoms and SARS-CoV-2 testing. Participants with ongoing severe COVID-19 or serious residual symptoms might not yet have completed the REGARDS study COVID-19 survey. Both mortality and severe COVID-19 are more common among Black than White individuals [5], which could have caused bias by race and SDOH. We did not have information about changes in residence during the pandemic which could potentially lead to misclassification of geographically defined SDOH. However, participants in REGARDS move infrequently; between study enrollment (2003–2007) and December 2017, 74% of the population had the same residential address. The survey did not contain questions about mask-wearing, physical distancing, or other infection risk mitigation strategies. There are many SDOH that we could not assess. In particular, we had limited information on social isolation, social networks, and living arrangements, which are important SDOH that have been impacted, sometimes dramatically, by the pandemic and mitigation efforts [40]. Finally, the pandemic has had complex geographic and temporal variation, which is reflected in the differences between responses in 2020 vs 2021. Because participants were asked to report experiences over the prior 6 months, we are not able to fully capture temporal variation. Additionally, we did not have sufficient sample size to examine variation at the scale of states or smaller geographic units. These limitations could obscure modification of the associations of race and SDOH with health care experiences by local pandemic conditions. However, the timing of survey administration was random with respect to race, SDOH, geography, and other participant characteristics so estimates reflect time-averaged experiences.

## Conclusion

In this population of older Black and White US adults participating in a long-term health research study, substantial proportions of participants with COVID-19 symptoms and those without symptoms who desired testing did not receive SARS-CoV-2 testing. These findings reflect barriers to ensuring optimal medical care for this population that need to be addressed as the COVID-19 pandemic unfolds and after the pandemic ends.

## Supplementary Information

**Additional file 1.** REGARDS Computer-Assisted Telephone Interview CATI – COVID-19. REGARDS COVID-19 module administered beginning July 6, 2020.

**Additional file 2.** Health care experiences during the COVID-19 pandemic by race and social determinants of health among adults age ≥ 58 years in the REGARDS Study.

## Data Availability

The datasets generated during and/or analysed during the current study are not publicly available due to participant privacy concerns. In order to abide by its obligations with NIH/NINDS and the IRB of the University of Alabama at Birmingham, REGARDS facilitates data sharing through formal data use agreements. Any investigator is welcome to request the REGARDS data and documentation through this process. Requests for data access may be sent to the REGARDS study at regardsadmin@uab.edu.

## Abbreviations

CATI: Computer-assisted telephone interview
IRB: Institutional review board
NIH/NINDS: National Institutes of Health National Institute of Neurologic Disorders and Stroke
REGARDS: REasons for Geographic And Racial Differences in Stroke
RUCA: Rural Urban Community Area
SDOH: Social determinants of health
US: United States

## References

[1] Bassett MT, Chen JT, Krieger N (2020). Variation in racial/ethnic disparities in COVID-19 mortality by age in the United States: a cross-sectional study. Plos Med.

[2] Price-Haywood EG, Burton J, Fort D, Seoane L (2020). Hospitalization and mortality among black patients and white patients with Covid-19. N Engl J Med.

[3] Yang TC, SWE Choi, Sun F. COVID-19 cases in US counties: roles of racial/ethnic density and residential segregation. Ethn Health. 2021;26(1):11–21. 10.1080/13557858.2020.1830036 33059471

[4] Rossen LM, Ahmad FB, Anderson RN, Branum AM, Du C, Krumholz HM, Li SX, Lin Z, Marshall A, Sutton PD (2021). Disparities in excess mortality associated with COVID-19 - United States, 2020. MMWR Morb Mortal Wkly Rep.

[5] Centers for Disease Control and Prevention. COVID-19 Hospitalization and Death by Race/Ethnicity. 2021. Available from: https://www.cdc.gov/coronavirus/2019-ncov/covid-data/investigations-discovery/hospitalization-death-by-race-ethnicity.html. Accessed 30 Sept 2021.

[6] Jones CP (2000). Levels of racism: a theoretic framework and a gardener’s tale. Am J Public Health.

[7] Garcia MA, Homan PA, Garcia C, Brown TH. The color of COVID-19: structural racism and the Pandemic's disproportionate impact on older racial and ethnic minorities. J Gerontol B Psychol Sci Soc Sci. 2021;76(3):e75–80. 10.1093/geronb/gbaa114 PMC7454923 32756973

[8] Bibbins-Domingo K (2020). This time must be different: disparities during the COVID-19 pandemic. Ann Intern Med.

[9] Tai DBG, Shah A, Doubeni CA, Sia IG, Wieland ML. The disproportionate impact of COVID-19 on racial and ethnic minorities in the United States. Clin Infect Dis. 2021;72(4):703–6. 10.1093/cid/ciaa815 PMC7337626 32562416

[10] Milam AJ, Furr-Holden D, Edwards-Johnson J, Webb B, Patton JW, Ezekwemba NC, Porter L, Davis T, Chukwurah M, Webb AJ (2020). Are clinicians contributing to excess African American COVID-19 deaths? Unbeknownst to them, they may be. Health Equity.

[11] Milner A, Franz B, Henry Braddock J (2020). We need to talk about racism-in all of its forms-to understand COVID-19 disparities. Health Equity.

[12] Office of Disease Prevention and Health Promotion, U.S. Department of Health and Human Services. Social Determinants of Health. https://www.healthypeople.gov/2020/topics-objectives/topic/social-determinants-of-health. Accessed 2 Dec 2020.

[13] Williams DR, Cooper LA (2020). COVID-19 and health equity—a new kind of “herd immunity”. JAMA.

[14] Tartof SY, Qian L, Hong V, Wei R, Nadjafi RF, Fischer H, Li Z, Shaw SF, Caparosa SL, Nau CL (2020). Obesity and mortality among patients diagnosed with COVID-19: results from an integrated health care organization. Ann Intern Med.

[15] Wadhera RK, Wadhera P, Gaba P, Figueroa JF, Joynt Maddox KE, Yeh RW, Shen C (2020). Variation in COVID-19 hospitalizations and deaths across new York City boroughs. JAMA.

[16] Palacio A, Tamariz L. Social determinants of health mediate COVID-19 disparities in South Florida. J Gen Intern Med. 2021;36(2):472–7. 10.1007/s11606-020-06341-9 PMC7673244 33206324

[17] Do DP, Frank R. Unequal burdens: assessing the determinants of elevated COVID-19 case and death rates in New York City’s racial/ethnic minority neighbourhoods. J Epidemiol Community Health. 2020:jech-2020-215280. 10.1136/jech-2020-215280. Epub ahead of print. 10.1136/jech-2020-215280 33122256

[18] Perry BL, Aronson B, Pescosolido BA. Pandemic precarity: COVID-19 is exposing and exacerbating inequalities in the American heartland. Proc Natl Acad Sci U S A. 2021;118(8):e2020685118. 10.1073/pnas.2020685118. 10.1073/pnas.2020685118 PMC7923675 33547252

[19] Jones CP. Coronavirus disease discriminates. Our Health Care Doesn't Have To. Newsweek. 2020. Available from: https://www.newsweek.com/2020/04/24/coronavirus-disease-discriminates-our-health-care-doesnt-have-opinion-1496405.html. Accessed 30 Sept 2021.

[20] Rentsch CT, Kidwai-Khan F, Tate JP, Park LS, King JT, Skanderson M, Hauser RG, Schultze A, Jarvis CI, Holodniy M (2020). Patterns of COVID-19 testing and mortality by race and ethnicity among United States veterans: a nationwide cohort study. PLoS Med.

[21] Escobar GJ, Adams AS, Liu VX, Soltesz L, Chen YI, Parodi SM, et al. Racial disparities in COVID-19 testing and outcomes: retrospective cohort study in an integrated health system. Ann Intern Med. 2021;174(6):786–93. 10.7326/M20-6979 PMC7893537 33556278

[22] Howard VJ, Cushman M, Pulley L, Gomez CR, Go RC, Prineas RJ, Graham A, Moy CS, Howard G (2005). The reasons for geographic and racial differences in stroke study: objectives and design. Neuroepidemiology.

[23] Howard VJ, Kleindorfer DO, Judd SE, McClure LA, Safford MM, Rhodes JD, Cushman M, Moy CS, Soliman EZ, Kissela BM (2011). Disparities in stroke incidence contributing to disparities in stroke mortality. Ann Neurol.

[24] Long DL, Howard G, Long DM, Judd S, Manly JJ, McClure LA, Wadley VG, Safford MM, Katz R, Glymour MM (2019). An investigation of selection Bias in estimating racial disparity in stroke risk factors. Am J Epidemiol.

[25] Sterling MR, Ringel JB, Pinheiro LC, Safford MM, Levitan EB, Phillips E, Brown TM, Goyal P (2020). Social determinants of health and 90-day mortality after hospitalization for heart failure in the REGARDS study. J Am Heart Assoc.

[26] Brooks MS, Bennett A, Lovasi GS, Hurvitz PM, Colabianchi N, Howard VJ, Manly J, Judd SE (2021). Matching participant address with public records database in a US national longitudinal cohort study. SSM Popul Health.

[27] Economic Research Service, US Department of Agriculture. Rural-Urban Community Area Codes. 2020. Available from: https://www.ers.usda.gov/data-products/rural-urban-commuting-area-codes.aspx. Accessed 22 Sept 2021.

[28] United Health Foundation. America’s Health Rankings. 2021. Available from: https://www.americashealthrankings.org/. Accessed 6 Sept 2021.

[29] Cummings DM, Patil SP, Long DL, Guo B, Cherrington A, Safford MM, Judd SE, Howard VJ, Howard G, Carson AP (2021). Does the association between hemoglobin A1c and risk of cardiovascular events vary by residential segregation? The REasons for geographic and racial differences in stroke (REGARDS) study. Diabetes Care.

[30] Safford MM, Brown TM, Muntner PM, Durant RW, Glasser S, Halanych JH, Shikany JM, Prineas RJ, Samdarshi T, Bittner VA (2012). Association of race and sex with risk of incident acute coronary heart disease events. JAMA.

[31] Pinheiro LC, Reshetnyak E, Sterling MR, Levitan EB, Safford MM, Goyal P (2020). Multiple vulnerabilities to health disparities and incident heart failure hospitalization in the REGARDS study. Circ Cardiovasc Qual Outcomes.

[32] United States Census Bureau. State Population by Characteristics: 2010–2020. 2021. Available from: https://www.census.gov/programs-surveys/popest/technical-documentation/research/evaluation-estimates/2020-evaluationestimates/2010s-state-detail.html. Accessed 26 Sept 2021.

[33] Centers for Disease Control and Prevention. Updated Guidance on Evaluating and Testing Persons for Coronavirus Disease 2019 (COVID-19) March 08, 2020. 2020. Available from: https://emergency.cdc.gov/han/2020/han00429.asp?deliveryName=USCDC_511-DM22015. Accessed 2 Dec 2020.

[34] Mody A, Pfeifauf K, Bradley C, Fox B, Hlatshwayo MG, Ross W, et al. Understanding drivers of COVID-19 racial disparities: a population-level analysis of COVID-19 testing among black and white populations. Clin Infect Dis. 2021;73(9):e2921–31. 10.1093/cid/ciaa1848 PMC7799327 33315066

[35] Gu T, Mack JA, Salvatore M, Prabhu Sankar S, Valley TS, Singh K, Nallamothu BK, Kheterpal S, Lisabeth L, Fritsche LG (2020). Characteristics associated with racial/ethnic disparities in COVID-19 outcomes in an academic health care system. JAMA Netw Open.

[36] Razjouyan J, Helmer DA, Li A, Naik AD, Amos CI, Bandi V, et al. Differences in COVID-19-related testing and healthcare utilization by race and ethnicity in the veterans health administration. J Racial Ethn Health Disparities. 2021:1–8. 10.1007/s40615-021-00982-0. Epub ahead of print. 10.1007/s40615-021-00982-0 PMC7945621 33694124

[37] Oran DP, Topol EJ. The proportion of SARS-CoV-2 infections that are asymptomatic: a systematic review. Ann Intern Med. 2021;174(5):655–62. 10.7326/M20-6976 PMC7839426 33481642

[38] Whaley CM, Pera MF, Cantor J, Chang J, Velasco J, Hagg HK, Sood N, Bravata DM (2020). Changes in health services use among commercially insured US populations during the COVID-19 pandemic. JAMA Netw Open.

[39] Mefford M, Safford MM, Muntner P, Durant RW, Brown TM, Levitan EB (2017). Insurance, self-reported medication adherence and LDL cholesterol: the REasons for geographic and racial differences in stroke study. Int J Cardiol.

[40] Gauthier GR, Smith JA, Garcia C, Garcia MA, Thomas PA. Exacerbating inequalities: social networks, racial/ethnic disparities, and the COVID-19 pandemic. J Gerontol B Psychol Sci Soc Sci. 2021;76(3):e88–92. 10.1093/geronb/gbaa117 PMC7454830 32756978

