# Supplementary material for: Health care experiences during the COVID-19 pandemic by race and social determinants of health among adults age ≥ 58 years in the REGARDS study
Source: BMC Public Health. 2021 Dec 11;21:2255. doi: 10.1186/s12889-021-12273-8 (PMC8665704; doi:10.1186/s12889-021-12273-8)
Supplement: Supplementary file 1 — Additional file 1. REGARDS Computer-Assisted Telephone Interview CATI – COVID-19. REGARDS COVID-19 module administered beginning July 6, 2020. [file 12889_2021_12273_MOESM1_ESM.pdf]

REGARDS Computer-Assisted Telephone Interview CATI – COVID-19 – Final programming REVISED 06 17 2020

<u>Add at end of regular 6-month call, right before asking for update on contact information</u>.

▪

CVD_ST
[Placeholder question to mark the beginning time of the COVID 19 module.]

CVDSTTM.
Capture Start time for COVID 19 module

<u>COVID 19 Script for REGARDS CATI follow-up:</u>
CVD_IN
The next few questions are regarding COVID-19 or coronavirus and how the pandemic might have affected your health. We've learned a great deal about how this virus affects older adults. However, we have more to learn.

Q1. In the past six months, have you been sick for more than one day with an illness that included any of the following: fever, cough, sore throat, chills, muscle pain, new loss of taste or smell, or shortness of breath or difficulty breathing? [INSTRUCTIONS: Try to get through reading all of the symptoms. Tell participant the next questions ask about each of the specific symptoms.]

1.	Yes	continue
2.	No	skip to Q2.
7.	Don't Know/Not Sure	skip to Q2.
9.	Refused	skip to Q2.

Q1.1. When did the symptoms start? [i.e., approximate date of onset]
(Probe for month and year)

___________	Enter date yyyy/ mm/dd
7777/01/01	Don't Know/Not Sure
9999/01/01	Refused

If respondent unable to remember exact day, enter the appropriate month and year and "01"
DATES ARE VERY IMPORTANT!!! AT THE VERY LEAST, GET MONTH and YEAR. PROBE USING LAST WEEK or LAST MONTH

Q1.2 Which of the following symptoms did you have? [INSTRUCTIONS – read each one. indicate that more than one answer is acceptable

1	Fever
2	Cough
3	Sore throat
4	Chills
5	Muscle Pain

6 New loss of taste or smell
7 Shortness of breath or difficulty breathing

[INSTRUCTIONS: Interviewer, please check that at least one of the symptoms is answered yes. If not, please review answer to Q1 with participant and ask which of those symptoms the patient had or else they may want to change their answer to Q1.]

8 Go to Q1 to review participant's answer skip to Q1,
this option is Exclusive to other options, meaning if any of the other options is selected then this will be grayed out and if this option is selected then all other options will be grayed out.

Q1.3 For this illness, did you seek advice from a healthcare professional?

1.	Yes	continue
2.	No	skip to Q2
7.	Don't Know/Not Sure	skip to Q2
9.	Refused	skip to Q2

Q1.4 Where did you seek advice or care? [INSTRUCTIONS: indicate that more than one answer is acceptable]

1 Doctor’s office
2 Telemedicine/telephone triage
3 Retail clinic/pharmacy
4 Urgent care
.5 Emergency Department
6 Health Department/Public Health clinic
7 Other – skip to Q1.5

Q.1.4.7.1 What was this other place? [text] ______________________
CVDQ1_4SP

Q1.5 How long after your symptoms started did you seek care? [INSTRUCTIONS: Read possible answers]

1. Less than 2 days
2. 2 to 7 days
3. Greater than a week
7. Don’t know/not sure
9. Refused

Q1.6 For this illness, were you tested for coronavirus (COVID-19)?

1.	Yes	continue
2.	No	skip to Q2.
7.	Don't Know/Not Sure	skip to Q2.
9.	Refused	skip to Q2.

Q1.6.1 Which test was used? [Instructions read the possible answers if necessary]

1. Nasal swab/swab in nose
2. Blood Draw
3. Other CVDQ1_6_1SP

Q1.6.1.1 What was this other test? [text]_____________________

Q1.6.2 Was the test for coronavirus positive?

1.	Yes
2.	No
7.	Don't Know/Not Sure
9.	Refused

Skip to Q3.

This next set of questions will be asked of persons who did not report symptoms in Question 1 and of persons who did have symptoms in Q1 but did not get tested.

Q2. At any time in the last 6 months, did you want to get a COVID19 test?

1.	Yes
2.	No
7.	Don't Know/Not Sure
9.	Refused

Q2.1 Did you get a COVID19 test?

1.	Yes	Continue
2.	No	Skip to Q3.
7.	Don't Know/Not Sure	Skip to Q3.
9.	Refused	Skip to Q3.

Q.2.2 Which test was used? [Instructions read the names of tests if necessary] Only one answer is allowed.

1. Nasal swab/swab in nose
2. Blood Draw
3. Other CVDQ2_2SP

Q2.2.1 What was this other test? [text]_____________________

Q2.3 Was the test for coronavirus positive?

1. Yes
2. No
7. Don't Know/Not Sure
9. Refused

This is for everyone to answer, whether or not they had symptoms

Q3. Whether or not you had a test, and whether the test was positive or negative, do you think you have had coronavirus (COVID-19)?

1. Yes
2. No
7. Don't Know/Not Sure
9. Refused

Q4: The next question is about how you feel the coronavirus pandemic has impacted your medical health care access. I will read possible answers.

0- No change
1- Mild. Appointments moved to telehealth.
2- Moderate. Delays or cancellations in appointments and/or delays in getting prescriptions; changes have minimal impact on health.
3- Severe. Unable to access needed care resulting in moderate to severe impact on health.

Q5: Interviewer: *Before we close, I would like to ask you one more question. We want to study how the COVID-19 / coronavirus pandemic is affecting REGARDS participants. One of the ways to do this is to test the blood for any evidence of previous infection. You might have heard about an antibody test. Sometimes people with an infection have no symptoms and sometimes people get very sick. The blood test will help us learn if you were infected with COVID-19 at some point. We would come to your home again for a very brief visit, shorter than past study visits. A health professional would take a blood sample. The health professional would wear protective equipment, like a mask, gloves and gown. To keep you safe, we would not come if you or a family member were sick. The visit would take about 10-15 minutes. This visit would happen this fall or early next year. We would mail you a $30 gift card like before. For planning, we are trying to see how many people may be interested in this. How likely do you think you would be to say yes if we asked you to let us come to your home and collect a blood sample?*

Would you say: [Interviewer: read the 4 possible responses]

Very likely (1)
Likely (2)
Unlikely (3)
Very unlikely (4)

Interviewer: Encourage response for what the participant feels now. Do not read but record if participant says:

8- Don't know/not sure
9- Refused to answer

CVDCOMMNT
Comments for COVID 19 module

Allow free text and record comments. We would like to work with SRU on a FAQ sheet but here is a start:

FAQ1: Participant might indicate he/she was already tested.
A1: Ask the participant what kind of test it was, nasal swab or blood test and record what type of test it was. Ask what the result was. Say to the participant: "Even though you were already tested, having this new test would allow us better understand the disease. Some people who test positive will not have continued evidence of this in their blood over time, and that might be important. If you tested negative in the past, it still could be that you were exposed to COVID-19, and we might find that.   There is a lot of work being done now to identity the most accurate tests." Then go back and ask them to still answer the question.

Q2: Will I get the results back?
A2: Yes, we assume so, but we have to work through that with the lab we select.

Q3: I've already told you about my COVID 19 hospital admission?
A3: Yes you did, please forgive us for any duplications and thank you for answering these detailed questions information about how COVID affected you.

CVDEND
END OF SURVEY

CVDENTM.
Capture End time for COVID 19 module.

CVDTIME.
Total time for COVID 19 module

CVDFLG.
Computed question to act as COVID 19 module's flag. If COVID19 module has been asked twice on consecutive sequences then the respondent should never be asked again.
