# Supplementary material for: Health care experiences during the COVID-19 pandemic by race and social determinants of health among adults age ≥ 58 years in the REGARDS study
Source: BMC Public Health. 2021 Dec 11;21:2255. doi: 10.1186/s12889-021-12273-8 (PMC8665704; doi:10.1186/s12889-021-12273-8)
Supplement: Supplementary file 2 — Additional file 2. Health care experiences during the COVID-19 pandemic by race and social determinants of health among adults age ≥ 58 years in the REGARDS Study. [file 12889_2021_12273_MOESM2_ESM.pdf]

SUPPLEMENTAL MATERIAL

Health care experiences during the COVID-19 pandemic by race and social determinants of health among adults age ≥58 years in the REGARDS Study

Emily B. Levitan,[1] Virginia J. Howard,[1] Mary Cushman,[2] Suzanne E. Judd,[1] Stephanie E. Tison,[1] Ya Yuan,[1] Debora Kamin Mukaz,[2] Henry E Wang,[3] Nathalie Pamir,[4] Timothy Plante,[2] Stephen P. Juraschek,[5] Monika M. Safford,[6] Parag Goyal[6]

[1]University of Alabama at Birmingham, Birmingham AL

[2]University of Vermont, Burlington, VT

[3]University of Texas, Houston, TX

[4]Oregon Health and Science University, Portland, OR

[5]Beth Israel Deaconess Medical Center, Boston, MA

[6]Weill Cornell Medicine, New York, NY

Correspondence to Emily B. Levitan, ScD, Department of Epidemiology, University of Alabama at Birmingham, Birmingham, AL 35294-0022, 205-975-7680, elevitan@uab.edu

Figure 1. Logic of the REGARDS study COVID-19 survey module[a]

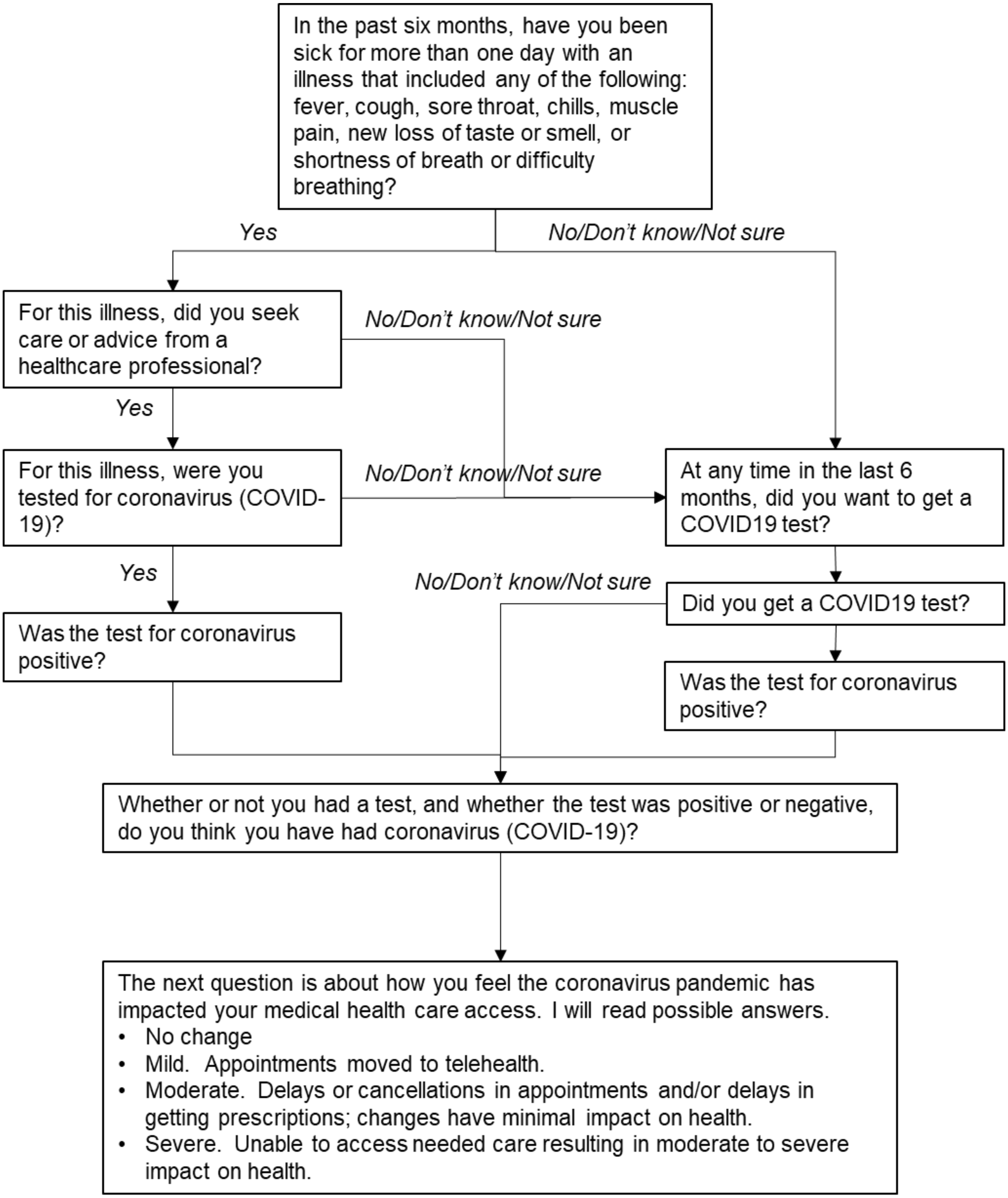

[a]Selected survey items

Table 1. Race, social determinants of health, and SARS-CoV-2 testing among participants in the REasons for Geographic And Racial Differences in Stroke (REGARDS) study who reported illness with symptoms of COVID-19[a]

		Sought care or advice		SARS-CoV-2 test		Positive SARS-CoV-2 test	
	N	N (%[a])	p-value[b]	N (%[a])	p-value[b]	N (%[a])	p-value[b]
Overall	697	514 (73.7%)		349 (50.1%)		173 (24.8%)	
Black	238	177 (74.4%)	0.85	126 (52.9%)	0.28	66 (27.7%)	0.20
White	459	337 (73.4%)		223 (48.6%)		107 (23.3%)	
Income <$35,000	216	159 (73.6%)	0.97	107 (49.5%)	0.97	53 (24.5%)	0.88
Income ≥$35,000	402	296 (73.6%)		203 (50.5%)		102 (25.4%)	
Income not reported	79	59 (74.7%)		39 (49.4%)		18 (22.8%)	
Unmarried	257	184 (71.6%)	0.31	126 (49.0%)	0.67	54 (21.0%)	0.08
Married	440	330 (75.0%)		223 (50.7%)		119 (27.1%)	
Less than high school	39	25 (64.1%)	0.33	14 (35.9%)	0.07	9 (23.1%)	0.80
High school and greater	658	489 (74.3%)		335 (50.9%)		164 (24.9%)	
Area-level poverty >25%	116	87 (75.0%)	0.77	61 (52.6%)	0.46	37 (31.9%)	0.047
Area-level poverty ≤25%	565	414 (73.3%)		276 (48.9%)		131 (23.2%)	
Rural	133	110 (82.7%)	0.02	83 (62.4%)	0.002	46 (34.6%)	0.004
Urban	551	398 (72.2%)		262 (47.6%)		124 (22.5%)	
Poor public health infrastructure	246	189 (76.8%)	0.26	145 (58.9%)	<0.001	75 (30.5%)	0.01
Better public health infrastructure	451	325 (72.1%)		204 (45.2%)		98 (21.7%)	
Medicaid non-expansion	372	276 (74.2%)	0.96	192 (51.6%)	0.38	102 (27.4%)	0.09
Medicaid expansion	325	238 (73.2%)		157 (48.3%)		71 (21.9%)	
More segregated neighborhood	323	242 (74.9%)	0.37	161 (49.9%)	0.88	72 (22.3%)	0.15
Less segregated neighborhood	373	272 (72.9%)		188 (50.4%)		101 (27.1%)	

[a]Row percentage. Denominator is number of individuals with that characteristic.

[b]Chi-square test comparing percentage with versus without the characteristic

Table 1a. Race, social determinants of health, and SARS-CoV-2 testing among participants in the REasons for Geographic And Racial Differences in Stroke (REGARDS) study who reported illness with symptoms of COVID-19, standardized to the age, race, sex, and geographic distribution of estimated US 2020 population

	Sought care or advice		SARS-CoV-2 test		Positive SARS-CoV-2 test	
	%[a]	p-value[b]	%[a]	p-value[b]	%[a]	p-value[b]
Overall	71.1%		45.3%		21.3%	
Black	76.3%	0.31	58.1%	0.02	34.2%	0.007
White	70.5%		43.9%		19.9%	
Income <$35,000	71.9%	0.87	41.6%	0.33	22.6%	0.89
Income ≥$35,000	71.9%		47.7%		21.3%	
Income not reported	63.3%		35.9%		18.0%	
Unmarried	64.6%	0.15	37.8%	0.11	14.7%	0.07
Married	74.0%		48.6%		24.2%	
Less than high school	59.6%	0.45	43.0%	0.88	34.8%	0.33
High school and greater	71.5%		45.4%		20.8%	
Area-level poverty >25%	56.2%	0.11	45.0%	0.96	36.3%	0.06
Area-level poverty ≤25%	72.4%		44.5%		19.5%	
Rural	77.5%	0.46	54.1%	0.27	33.1%	0.046
Urban	71.3%		44.3%		18.8%	
Poor public health infrastructure	75.5%	0.42	57.0%	0.051	23.0%	0.68
Better public health infrastructure	70.0%		42.5%		20.9%	
Medicaid non-expansion	70.8%	0.94	52.2%	0.15	25.3%	0.29
Medicaid expansion	71.2%		42.9%		19.9%	
More segregated neighborhood	71.2%	0.98	42.6%	0.45	19.8%	0.61
Less segregated neighborhood	71.0%		47.6%		22.5%	

[a]Row percentage standardized to the age, race, sex, and geographic distribution of estimated US 2020 population

[b]P-value from bivariate logistic regression with inverse probability of sampling weights and robust standard errors

Table 1b. Race, social determinants of health, and SARS-CoV-2 testing among participants in the REasons for Geographic And Racial Differences in Stroke (REGARDS) study who reported illness with symptoms of COVID-19, July 6, 2020-December 31, 2020

		Sought care or advice		SARS-CoV-2 test		Positive SARS-CoV-2 test	
	N	N (%[a])	p-value[b]	N (%[a])	p-value[b]	N (%[a])	p-value[b]
Overall	506	367 (72.5%)		221 (43.7%)		83 (16.4%)	
Black	166	199 (71.7%)	0.77	81 (48.8%)	0.10	37 (22.3%)	0.01
White	340	248 (72.9%)		140 (41.2%)		46 (13.5%)	
Income <$35,000	152	113 (74.3%)	0.83	66 (43.4%)	0.87	24 (15.8%)	0.53
Income ≥$35,000	295	212 (71.9%)		131 (44.4%)		52 (17.6%)	
Income not reported	59	42 (71.2%)		24 (40.7%)		7 (11.9%)	
Unmarried	187	137 (73.3%)	0.78	84 (44.9%)	0.67	30 (16.0%)	0.87
Married	319	230 (72.1%)		137 (42.9%)		53 (16.6%)	
Less than high school	26	16 (61.5%)	0.20	6 (23.1%)	0.03	2 (7.7%)	0.22
High school and greater	480	351 (73.1%)		215 (44.8%)		81 (16.9%)	
Area-level poverty >25%	79	56 (70.9%)	0.75	34 (43.0%)	0.94	21 (26.6%)	0.005
Area-level poverty ≤25%	413	300 (72.6%)		176 (42.6%)		58 (14.0%)	
Rural	90	75 (83.3%)	0.02	52 (57.8%)	0.004	22 (24.4%)	0.02
Urban	407	288 (70.8%)		167 (41.0%)		59 (14.5%)	
Poor public health infrastructure	181	134 (74.0%)	0.57	94 (51.9%)	0.005	39 (21.5%)	0.02
Better public health infrastructure	325	233 (71.7%)		127 (39.1%)		44 (13.5%)	
Medicaid non-expansion	266	192 (72.2%)	0.85	121 (45.5%)	0.39	49 (18.4%)	0.20
Medicaid expansion	240	175 (72.9%)		100 (41.7%)		34 (14.2%)	
More segregated neighborhood	244	179 (73.4%)	0.74	107 (43.9%)	0.97	37 (15.2%)	0.46
Less segregated neighborhood	261	188 (72.0%)		114 (43.7%)		46 (17.6%)	

[a]Row percentage. Denominator is number of individuals with that characteristic.

[b]Chi-square test comparing percentage with versus without the characteristic

Table 1c. Race, social determinants of health, and SARS-CoV-2 testing among participants in the REasons for Geographic And Racial Differences in Stroke (REGARDS) study who reported illness with symptoms of COVID-19, January 1, 2021-September 4, 2021

		Sought care or advice		SARS-CoV-2 test		Positive SARS-CoV-2 test	
	N	N (%[a])	p-value[b]	N (%[a])	p-value[b]	N (%[a])	p-value[b]
Overall	191	147 (77.0%)		128 (67.0%)		90 (47.1%)	
Black	72	58 (80.6%)	0.57	45 (62.5%)	0.30	29 (40.3%)	0.14
White	119	89 (74.8%)		83 (69.7%)		61 (51.3%)	
Income <$35,000	64	46 (71.9%)	0.74	41 (64.1%)	0.66	29 (45.3%)	0.74
Income ≥$35,000	107	84 (78.5%)		72 (67.3%)		50 (46.7%)	
Income not reported	20	17 (85.0%)		15 (75.0%)		11 (55.0%)	
Unmarried	70	47 (67.1%)	0.01	42 (60.0%)	0.12	24 (34.3%)	0.007
Married	121	100 (82.6%)		86 (71.1%)		66 (54.5%)	
Less than high school	13	9 (69.2%)	0.69	8 (61.5%)	0.66	7 (53.8%)	0.61
High school and greater	178	138 (77.5%)		120 (67.4%)		83 (46.6%)	
Area-level poverty >25%	37	31 (83.8%)	0.46	27 (73.0%)	0.40	16 (43.2%)	0.60
Area-level poverty ≤25%	152	114 (75.0%)		100 (65.8%)		73 (48.0%)	
Rural	43	35 (81.4%)	0.45	31 (72.1%)	0.45	24 (55.8%)	0.22
Urban	144	110 (76.4%)		95 (66.0%)		65 (45.1%)	
Poor public health infrastructure	65	55 (84.6%)	0.15	51 (78.5%)	0.02	36 (55.4%)	0.10
Better public health infrastructure	126	92 (73.0%)		77 (61.1%)		54 (42.9%)	
Medicaid non-expansion	106	84 (79.2%)	0.70	71 (67.0%)	0.99	53 (50.0%)	0.37
Medicaid expansion	85	63 (74.1%)		57 (67.1%)		37 (43.5%)	
More segregated neighborhood	79	63 (79.7%)	0.42	54 (68.4%)	0.74	35 (44.3%)	0.51
Less segregated neighborhood	112	84 (75.0%)		74 (66.1%)		55 (49.1%)	

[a]Row percentage. Denominator is number of individuals with that characteristic.

[b]Chi-square test comparing percentage with versus without the characteristic

Table 2. Race, social determinants of health, and SARS-CoV-2 testing among participants in the REasons for Geographic And Racial Differences in Stroke (REGARDS) study who did not report illness with symptoms of COVID-19

		Wanted a SARS-CoV-2 test		Wanted and received a SARS-CoV-2 test		Did not want but received a SARS-CoV-2 test		Positive SARS-CoV-2 test	
	N	N (%[a])	p-value[b]	N (%[a])	p-value[b]	N (%[a])	p-value[b]	N (%[a])	p-value[b]
Overall	8,795	2,525 (28.7%)		1,972 (22.4%)		744 (8.5%)		85 (1.0%)	
Black	3,178	1,222 (38.5%)	<0.001	948 (29.8%)	<0.001	280 (8.8%)	0.37	43 (1.4%)	0.005
White	5,617	1,303 (23.2%)		1,024 (18.2%)		464 (8.3%)		42 (0.8%)	
Income <$35,000	2,660	777 (29.2%)	0.01	580 (21.8%)	0.009	222 (8.4%)	0.46	37 (1.4%)	0.03
Income ≥$35,000	4,954	1,451 (29.3%)		1,162 (23.5%)		432 (8.7%)		40 (0.8%)	
Income not reported	1,181	297 (25.2%)		230 (19.5%)		90 (7.6%)		8 (0.7%)	
Unmarried	3,468	1,057 (30.5%)	0.003	818 (23.6%)	0.03	295 (8.5%)	0.90	38 (1.1%)	0.32
Married	5,327	1,468 (27.6%)		1,154 (21.7%)		449 (8.4%)		47 (0.9%)	
Less than high school	519	145 (27.9%)	0.69	112 (21.6%)	0.63	48 (9.3%)	0.51	12 (2.3%)	0.001
High school and greater	8,275	2,380 (28.8%)		1,860 (22.5%)		696 (8.4%)		73 (0.9%)	
Area-level poverty >25%	1,372	470 (34.3%)	<0.001	365 (26.6%)	<0.001	118 (8.6%)	0.77	18 (1.3%)	0.16
Area-level poverty ≤25%	7,273	2,018 (27.8%)		1,576 (21.7%)		608 (8.4%)		66 (0.9%)	
Rural	1,451	375 (25.8%)	0.008	319 (22.0%)	0.66	122 (8.4%)	0.96	14 (1.0%)	0.96
Urban	7,256	2,126 (29.3%)		1,633 (22.5%)		613 (8.5%)		71 (1.0%)	
Poor public health infrastructure	2,890	834 (28.9%)	0.83	660 (22.8%)	0.51	229 (7.9%)	0.21	31 (1.1%)	0.48
Better public health infrastructure	5,905	1,691 (28.6%)		1,312 (22.2%)		515 (8.7%)		54 (0.9%)	
Medicaid non-expansion	4,759	1,339 (28.1%)	0.20	1,060 (22.3%)	0.72	385 (8.1%)	0.18	51 (1.1%)	0.27
Medicaid expansion	4,036	1,186 (29.4%)		912 (22.6%)		359 (8.9%)		34 (0.8%)	
More segregated neighborhood	4,421	1,402 (31.7%)	<0.001	1,084 (24.5%)	<0.001	383 (8.7%)	0.48	49 (1.1%)	0.17
Less segregated neighborhood	4,368	1,123 (25.7%)		888 (20.3%)		360 (8.2%)		36 (0.8%)	

[a]Row percentage. Denominator is number of individuals with that characteristic.

[b]Chi-square test comparing percentage with versus without the characteristic

Table 2a. Race, social determinants of health, and SARS-CoV-2 testing among participants in the REasons for Geographic And Racial Differences in Stroke (REGARDS) study who did not report illness with symptoms of COVID-19, standardized to the age, race, sex, and geographic distribution of the 2020 US Census

	Wanted a SARS-CoV-2 test		Wanted and received a SARS-CoV-2 test		Did not want but received a SARS-CoV-2 test		Positive SARS-CoV-2 test	
	%[a]	p-value[b]	%[a]	p-value[b]	%[a]	p-value[b]	%[a]	p-value[b]
Overall	27.4%		21.3%		9.0%		0.6%	
Black	41.8%	<0.001	33.3%	<0.001	9.8%	0.44	1.2%	0.054
White	25.3%		19.6%		8.9%		0.6%	
Income <$35,000	28.0%	0.68	21.0%	0.40	8.4%	0.23	0.7%	0.94
Income ≥$35,000	28.0%		21.9%		9.6%		0.7%	
Income not reported	22.4%		17.8%		6.7%		0.3%	
Unmarried	30.1%	0.03	23.2%	0.09	9.0%	0.94	0.5%	0.58
Married	26.2%		20.4%		9.1%		0.7%	
Less than high school	17.9%	0.002	14.5%	0.01	10.3%	0.65	1.3%	0.12
High school and greater	27.6%		21.5%		9.0%		0.6%	
Area-level poverty >25%	34.4%	0.004	26.4%	0.02	9.6%	0.67	0.7%	0.71
Area-level poverty ≤25%	27.0%		21.0%		8.9%		0.6%	
Rural	20.0%	<0.001	16.9%	0.02	8.5%	0.66	0.4%	0.39
Urban	28.6%		21.9%		9.1%		0.7%	
Poor public health infrastructure	28.5%	0.46	22.8%	0.30	7.3%	0.06	0.8%	0.56
Better public health infrastructure	27.1%		20.9%		9.4%		0.6%	
Medicaid non-expansion	27.9%	0.67	22.4%	0.31	7.5%	0.03	0.6%	0.85
Medicaid expansion	27.2%		20.8%		9.7%		0.6%	
More segregated neighborhood	30.0%	0.002	23.2%	0.01	9.3%	0.59	0.6%	0.97
Less segregated neighborhood	24.7%		19.3%		8.7%		0.6%	

[a]Row percentage standardized to the age, race, sex, and geographic distribution of estimated US 2020 population

[b]P-value from bivariate logistic regression with inverse probability of sampling weights and robust standard errors

Table 2b. Race, social determinants of health, and SARS-CoV-2 testing among participants in the REasons for Geographic And Racial Differences in Stroke (REGARDS) study who did not report illness with symptoms of COVID-19, July 6, 2020-December 31, 2020

		Wanted a SARS-CoV-2 test		Wanted and received a SARS-CoV-2 test		Did not want but received a SARS-CoV-2 test		Positive SARS-CoV-2 test	
	N	N (%[a])	p-value[b]	N (%[a])	p-value[b]	N (%[a])	p-value[b]	N (%[a])	p-value[b]
Overall	6,561	1,709 (26.0%)		1,309 (20.0%)		515 (7.9%)		51 (0.8%)	
Black	2,304	827 (35.9%)	<0.001	635 (27.6%)	<0.001	185 (8.0%)	0.69	22 (1.0%)	0.23
White	4,257	882 (20.7%)		674 (15.8%)		330 (7.8%)		29 (0.7%)	
Income <$35,000	1,963	514 (26.2%)	0.04	376 (19.2%)	0.02	142 (7.2%)	0.36	24 (1.2%)	0.02
Income ≥$35,000	3,718	996 (26.8%)		783 (21.1%)		307 (8.3%)		23 (0.6%)	
Income not reported	880	199 (22.6%)		150 (17.0%)		66 (7.5%)		4 (0.5%)	
Unmarried	2,573	711 (27.6%)	0.02	542 (21.1%)	0.07	206 (8.0%)	0.70	24 (0.9%)	0.25
Married	3,988	998 (25.0%)		767 (19.2%)		309 (7.7%)		27 (0.7%)	
Less than high school	377	92 (24.4%)	0.45	69 (18.3%)	0.41	32 (8.5%)	0.64	9 (2.4%)	<0.001
High school and greater	6,183	1,617 (26.2%)		1,240 (20.1%)		483 (7.8%)		42 (0.7%)	
Area-level poverty >25%	1,001	318 (31.8%)	<0.001	245 (24.5%)	<0.001	81 (8.1%)	0.74	10 (1.0%)	0.42
Area-level poverty ≤25%	5,446	1,366 (25.1%)		1,044 (19.2%)		424 (7.8%)		41 (0.8%)	
Rural	1,086	251 (23.1%)	0.02	213 (19.6%)	0.77	87 (8.0%)	0.84	6 (0.6%)	0.34
Urban	5,415	1,442 (26.6%)		1,083 (20.0%)		424 (7.8%)		45 (0.8%)	
Poor public health infrastructure	2,181	571 (26.2%)	0.86	443 (20.3%)	0.61	164 (7.5%)	0.48	17 (0.8%)	0.99
Better public health infrastructure	4,380	1,138 (26.0%)		866 (19.8%)		351 (8.0%)		34 (0.8%)	
Medicaid non-expansion	3,519	898 (25.5%)	0.29	696 (19.8%)	0.71	263 (7.5%)	0.22	26 (0.7%)	0.70
Medicaid expansion	3,042	811 (26.7%)		613 (20.2%)		252 (8.3%)		25 (0.8%)	
More segregated neighborhood	3,299	948 (28.7%)	<0.001	716 (21.7%)	<0.001	265 (8.0%)	0.56	30 (0.9%)	0.22
Less segregated neighborhood	3,256	761 (23.4%)		593 (18.2%)		249 (7.6%)		21 (0.6%)	

[a]Row percentage. Denominator is number of individuals with that characteristic.

[b]Chi-square test comparing percentage with versus without the characteristic

Table 2c. Race, social determinants of health, and SARS-CoV-2 testing among participants in the REasons for Geographic And Racial Differences in Stroke (REGARDS) study who did not report illness with symptoms of COVID-19, January 1, 2021-September 6, 2021

		Wanted a SARS-CoV-2 test		Wanted and received a SARS-CoV-2 test		Did not want but received a SARS-CoV-2 test		Positive SARS-CoV-2 test	
	N	N (%[a])	p-value[b]	N (%[a])	p-value[b]	N (%[a])	p-value[b]	N (%[a])	p-value[b]
Overall	2,234	816 (36.5%)		663 (29.7%)		229 (10.3%)		34 (1.5%)	
Black	874	395 (45.2%)	<0.001	313 (35.8%)	<0.001	95 (10.9%)	0.44	21 (2.4%)	0.006
White	1,360	421 (31.0%)		350 (25.7%)		134 (9.9%)		13 (1.0%)	
Income <$35,000	697	263 (37.7%)	0.28	204 (29.3%)	0.36	80 (11.5%)	0.24	13 (1.9%)	0.67
Income ≥$35,000	1,236	455 (36.8%)		379 (30.7%)		125 (10.1%)		17 (1.4%)	
Income not reported	301	98 (32.6%)		80 (26.6%)		24 (8.0%)		4 (1.3%)	
Unmarried	895	346 (38.7%)	0.09	276 (30.8%)	0.33	89 (9.9%)	0.70	14 (1.6%)	0.89
Married	1,339	470 (35.1%)		387 (28.9%)		140 (10.5%)		20 (1.5%)	
Less than high school	142	53 (37.3%)	0.84	43 (30.3%)	0.87	16 (11.3%)	0.68	3 (2.1%)	0.55
High school and greater	2,092	763 (36.5%)		620 (29.6%)		213 (10.2%)		31 (1.5%)	
Area-level poverty >25%	371	152 (41.0%)	0.054	120 (32.3%)	0.21	37 (10.0%)	0.95	8 (2.2%)	0.26
Area-level poverty ≤25%	1,827	652 (35.7%)		532 (29.1%)		184 (10.1%)		25 (1.4%)	
Rural	365	124 (34.0%)	0.25	106 (29.0%)	0.75	35 (9.6%)	0.70	8 (2.2%)	0.27
Urban	1,841	684 (37.2%)		550 (29.9%)		189 (10.3%)		26 (1.4%)	
Poor public health infrastructure	709	263 (37.1%)	0.70	217 (30.6%)	0.51	65 (9.2%)	0.25	14 (2.0%)	0.23
Better public health infrastructure	1,525	553 (36.3%)		446 (29.2%)		164 (10.8%)		20 (1.3%)	
Medicaid non-expansion	1,240	441 (35.6%)	0.29	364 (29.4%)	0.71	122 (9.8%)	0.47	25 (2.0%)	0.03
Medicaid expansion	994	375 (37.7%)		299 (30.1%)		107 (10.8%)		9 (0.9%)	
More segregated neighborhood	1,122	454 (40.5%)	<0.001	368 (32.8%)	0.001	118 (10.5%)	0.68	19 (1.7%)	0.51
Less segregated neighborhood	1,112	362 (32.6%)		295 (26.5%)		111 (10.0%)		15 (1.3%)	

[a]Row percentage. Denominator is number of individuals with that characteristic.

[b]Chi-square test comparing percentage with versus without the characteristic

Table 3. Social determinants of health and impact of the COVID-19 pandemic on access to care among participants in the REasons for Geographic And Racial Differences in Stroke (REGARDS) study

	No change N (%[a])	Mild. Appointments moved to telehealth N (%[a])	Moderate. Delays or cancellations in appointments and/or delays in getting prescriptions; changes have minimal impact on health N (%[a])	Severe. Unable to access needed care resulting in moderate to severe impact on health N (%[a])	p-value[b]
Overall	4,840 (51.0%)	2,130 (22.4%)	2,381 (25.1%)	141 (1.5%)	
Black	1,817 (53.2%)	748 (21.9%)	802 (23.5%)	49 (1.4%)	0.01
White	3,023 (49.8%)	1,382 (22.8%)	1,579 (26.0%)	92 (1.5%)	
Income <$35,000	1,643 (57.1%)	532 (18.5%)	658 (22.9%)	43 (1.5%)	<0.001
Income ≥$35,000	2,477 (46.3%)	1,372 (25.6%)	1,431 (26.7%)	76 (1.4%)	
Income not reported	720 (57.1%)	226 (17.9%)	292 (23.2%)	22 (1.8%)	
Unmarried	1,970 (52.9%)	773 (20.8%)	922 (24.8%)	60 (1.6%)	0.005
Married	2,870 (49.8%)	1,357 (23.5%)	1,459 (25.3%)	81 (1.4%)	
Less than high school	390 (69.9%)	61 (10.9%)	98 (17.6%)	9 (1.6%)	<0.001
High school and greater	4,449 (49.8%)	2,069 (23.2%)	2,283 (25.6%)	132 (1.5%)	
Area-level poverty >25%	816 (54.8%)	322 (21.6%)	332 (22.3%)	18 (1.2%)	0.006
Area-level poverty ≤25%	3,930 (50.1%)	1,774 (22.6%)	2,014 (25.7%)	120 (1.5%)	
Rural	898 (56.7%)	325 (20.5%)	336 (21.2%)	25 (1.6%)	<0.001
Urban	3,889 (49.8%)	1,782 (22.8%)	2,020 (25.9%)	116 (1.5%)	
Poor public health infrastructure	1,696 (54.1%)	683 (21.8%)	711 (22.7%)	46 (1.5%)	<0.001
Better public health infrastructure	3,144 (49.5%)	1,447 (22.8%)	1,670 (26.3%)	95 (1.5%)	
Medicaid non-expansion	2,710 (52.8%)	1,111 (21.7%)	1,239 (24.2%)	71 (1.4%)	0.002
Medicaid expansion	2,130 (48.8%)	1,019 (23.4%)	1,142 (26.2%)	70 (1.6%)	
More segregated neighborhood	2,385 (50.3%)	1,039 (21.9%)	1,249 (26.3%)	71 (1.5%)	0.04
Less segregated neighborhood	2,451 (51.7%)	1,090 (23.0%)	1,130 (23.8%)	70 (1.5%)	

[a]Row percentage. Denominator is number of individuals with that characteristic.

[b]Chi-square test comparing percentage with versus without the characteristic

Table 3a. Social determinants of health and impact of the COVID-19 pandemic on access to care among participants in the REasons for Geographic And Racial Differences in Stroke (REGARDS) study, standardized to the age, race, sex, and geographic distribution of the 2020 US Census

	No change	Mild. Appointments moved to telehealth	Moderate. Delays or cancellations in appointments and/or delays in getting prescriptions; changes have minimal impact on health	Severe. Unable to access needed care resulting in moderate to severe impact on health	p-value[b]
	%[a]	%[a]	%[a]	%[a]	
Overall	41.3%	27.2%	29.8%	1.7%	
Black	43.4%	27.2%	27.4%	1.9%	0.33
White	41.0%	27.2%	30.1%	1.7%	
Income <$35,000	49.3%	21.6%	27.4%	1.7%	<0.001
Income ≥$35,000	37.9%	29.9%	30.6%	1.6%	
Income not reported	47.5%	20.8%	29.2%	2.4%	
Unmarried	44.4%	25.1%	28.8%	1.7%	0.12
Married	40.0%	28.1%	30.3%	1.7%	
Less than high school	55.8%	8.4%	34.7%	1.2%	<0.001
High school and greater	40.9%	27.7%	29.7%	1.7%	
Area-level poverty >25%	43.9%	26.1%	28.5%	1.6%	0.72
Area-level poverty ≤25%	40.8%	27.4%	30.1%	1.7%	
Rural	47.5%	25.5%	24.7%	2.3%	0.02
Urban	40.2%	27.4%	30.8%	1.6%	
Poor public health infrastructure	46.4%	24.0%	27.8%	1.8%	0.02
Better public health infrastructure	40.1%	27.9%	30.3%	1.7%	
Medicaid non-expansion	45.7%	24.7%	28.2%	1.5%	0.01
Medicaid expansion	39.6%	28.2%	30.5%	1.8%	
More segregated neighborhood	41.2%	25.3%	32.2%	1.4%	0.02
Less segregated neighborhood	41.5%	29.1%	27.4%	2.0%	

[a]Row percentage standardized to the age, race, sex, and geographic distribution of estimated US 2020 population

[b]P-value from multinomial logistic regression with inverse probability of sampling weights and robust standard errors

Table 3b. Social determinants of health and impact of the COVID-19 pandemic on access to care among participants in the REasons for Geographic And Racial Differences in Stroke (REGARDS) study, July 6, 2020-December 31, 2020

	No change N (%[a])	Mild. Appointments moved to telehealth N (%[a])	Moderate. Delays or cancellations in appointments and/or delays in getting prescriptions; changes have minimal impact on health N (%[a])	Severe. Unable to access needed care resulting in moderate to severe impact on health N (%[a])	p-value[b]
Overall	3,478 (49.2%)	1,640 (23.2%)	1,834 (26.0%)	115 (1.6%)	
Black	1,255 (50.8%)	574 (23.2%)	601 (24.3%)	40 (1.6%)	0.12
White	2,223 (48.4%)	1,066 (23.2%)	1,233 (26.8%)	75 (1.6%)	
Income <$35,000	1,170 (55.3%)	400 (18.9%)	508 (24.0%)	37 (1.7%)	<0.001
Income ≥$35,000	1,792 (44.7%)	1,066 (26.6%)	1,096 (27.3%)	59 (1.5%)	
Income not reported	516 (55.0%)	174 (18.5%)	230 (24.5%)	19 (2.0%)	
Unmarried	1,388 (50.3%)	592 (21.4%)	727 (26.3%)	53 (1.9%)	0.02
Married	2,090 (48.5%)	1,048 (24.3%)	1,107 (25.7%)	62 (1.4%)	
Less than high school	286 (71.0%)	42 (10.4%)	67 (16.6%)	8 (2.0%)	<0.001
High school and greater	3,191 (47.9%)	1,598 (24.0%)	1,767 (26.5%)	107 (1.6%)	
Area-level poverty >25%	576 (53.3%)	245 (22.7%)	246 (22.8%)	13 (1.2%)	0.01
Area-level poverty ≤25%	2,835 (48.4%)	1,367 (23.3%)	1,558 (26.6%)	99 (1.7%)	
Rural	645 (54.8%)	247 (21.0%)	264 (22.4%)	20 (1.7%)	<0.001
Urban	2,794 (48.0%)	1,377 (23.7%)	1,556 (26.7%)	95 (1.6%)	
Poor public health infrastructure	1,229 (52.0%)	540 (22.9%)	554 (23.5%)	39 (1.7%)	0.002
Better public health infrastructure	2,249 (47.8%)	1,100 (23.4%)	1,280 (27.2%)	76 (1.6%)	
Medicaid non-expansion	1,927 (50.9%)	850 (22.5%)	952 (25.2%)	56 (1.5%)	0.02
Medicaid expansion	1,551 (47.3%)	790 (24.1%)	882 (26.9%)	59 (1.8%)	
More segregated neighborhood	1,720 (48.5%)	803 (22.7%)	962 (27.2%)	58 (1.6%)	0.14
Less segregated neighborhood	1,754 (49.9%)	836 (23.8%)	870 (24.7%)	57 (1.6%)	

[a]Row percentage. Denominator is number of individuals with that characteristic.

[b]Chi-square test comparing percentage with versus without the characteristic

Table 3c. Social determinants of health and impact of the COVID-19 pandemic on access to care among participants in the REasons for Geographic And Racial Differences in Stroke (REGARDS) study, January 1, 2021-September 6, 2021

	No change N (%[a])	Mild. Appointments moved to telehealth N (%[a])	Moderate. Delays or cancellations in appointments and/or delays in getting prescriptions; changes have minimal impact on health N (%[a])	Severe. Unable to access needed care resulting in moderate to severe impact on health N (%[a])	p-value[b]
Overall	1,362 (56.2%)	490 (20.2%)	547 (22.6%)	26 (1.1%)	
Black	562 (59.4%)	174 (18.4%)	201 (21.2%)	9 (1.0%)	0.08
White	800 (54.1%)	316 (21.4%)	346 (23.4%)	17 (1.1%)	
Income <$35,000	473 (62.2%)	132 (17.3%)	150 (19.7%)	6 (0.8%)	<0.001
Income ≥$35,000	685 (51.0%)	306 (22.8%)	335 (24.9%)	17 (1.3%)	
Income not reported	204 (63.6%)	52 (16.2%)	62 (19.3%)	3 (0.9%)	
Unmarried	582 (60.3%)	181 (18.8%)	195 (20.2%)	7 (0.7%)	0.006
Married	780 (53.4%)	309 (21.2%)	352 (24.1%)	19 (1.3%)	
Less than high school	104 (67.1%)	19 (12.3%)	31 (20.0%)	1 (0.6%)	0.02
High school and greater	1,258 (55.4%)	471 (20.7%)	516 (22.7%)	25 (1.1%)	
Area-level poverty >25%	240 (58.8%)	77 (18.9%)	86 (21.1%)	5 (1.2%)	0.60
Area-level poverty ≤25%	1,095 (55.3%)	407 (20.6%)	456 (23.0%)	21 (1.1%)	
Rural	253 (62.0%)	78 (19.1%)	72 (17.6%)	5 (1.2%)	0.04
Urban	1,095 (55.2%)	405 (20.4%)	464 (23.4%)	21 (1.1%)	
Poor public health infrastructure	467 (60.3%)	143 (18.5%)	157 (20.3%)	7 (0.9%)	0.04
Better public health infrastructure	895 (54.2%)	347 (21.0%)	390 (23.6%)	19 (1.2%)	
Medicaid non-expansion	783 (58.2%)	261 (19.4%)	287 (21.3%)	15 (1.1%)	0.15
Medicaid expansion	579 (53.7%)	229 (21.2%)	260 (24.1%)	11 (1.0%)	
More segregated neighborhood	665 (55.4%)	236 (19.7%)	287 (23.9%)	13 (1.1%)	0.47
Less segregated neighborhood	697 (56.9%)	254 (20.8%)	260 (21.2%)	13 (1.1%)	

[a]Row percentage. Denominator is number of individuals with that characteristic.

[b]Chi-square test comparing percentage with versus without the characteristic
